# Misdiagnosis of Cerebellar Infarcts and Its Outcome

**DOI:** 10.7759/cureus.35362

**Published:** 2023-02-23

**Authors:** Ashwini Maria David, Aysha Jaleel, Chris Mariya Joy Mathew

**Affiliations:** 1 Internal Medicine, Jubilee Mission Medical College and Research Institute, Thrissur, IND; 2 Medicine, David Tvildiani Medical University, Tbilisi, GEO

**Keywords:** radiology, vertigo, stroke, misdiagnosis, ct brain, cerebellar infarct

## Abstract

Cerebellar infarction, a rare category of stroke, is often misdiagnosed but not given much importance in the available literature. Its presentation overlaps with symptoms of other neurologic, cardiovascular, gastrointestinal, and systemic conditions and therefore is nonspecific. Early diagnosis and management of cerebellar strokes are of utmost importance as the lack of a proper diagnosis may increase overall morbidity and mortality. Lack of awareness of the warning signs and symptoms, non-specificity of symptoms, absence of neurological deficits, and imaging discrepancies are some of the factors contributing to misdiagnosis and delayed treatment. If symptomatology is considered, it is found that symptoms of posterior circulation stroke were more frequently misdiagnosed compared to anterior circulation. Nausea and vomiting increased the chance further. Some other rare presentations include gastrointestinal symptoms, isolated vertigo, and symptoms of inner ear disease. Overdependence on radiological investigations often masks the significance of clinical examination. Ischemic stroke may appear normal in the initial 48 hours in the computed tomography scan of the brain or bony artefacts may hide the lesion. Permanent disabling deficits can follow a cerebellar stroke and the complications, which include hydrocephalus, brain stem compression, and gait abnormalities, necessitate prompt identification and management. In this review article, we aim at analysing various case reports of cerebellar infarction, the most common presentations that were under-evaluated, and their outcomes, thereby highlighting the importance of proper diagnosis and reporting of cerebellar infarction in the future. A thorough knowledge of the association between various clinical presentations of cerebellar stroke and its misdiagnosis helps clinicians to be more vigilant about the disease.

## Introduction and background

In the modern world, a change in lifestyle along with many other factors has contributed to a rapid rise in the occurrence of cerebrovascular diseases. According to global stroke estimates, 400-800 strokes occur per 100,000 population [1]. Cerebellar strokes, both ischemic and haemorrhagic, account for 2-3% of 600,000 strokes occurring annually in the United States and share a disproportionate level of morbidity and mortality. Cerebellar strokes have almost two times the mortality rate (23%) of cerebral strokes (12.5%), which are more common [2]. Atherosclerosis, other vasculopathies including arterial dissections, atrial fibrillation, or atrial flutter, and right-to-left shunt such as a patent foramen ovale increases the risk of cerebellar strokes along with the general risk factors for vascular disease such as age, smoking, obesity, diabetes, hyperlipidaemia, and hypertension [3,4]. The usual presenting symptoms in the case of a cerebellar stroke are dizziness, nausea, vomiting, slurred speech, gait incoordination, headache, and hearing loss [5]. Since these are non-specific symptoms, a thorough evaluation is necessary to aid the diagnosis.

Presentation of cerebellar infarction can overlap with the symptoms of other neurologic, cardiovascular, gastrointestinal, and other systemic conditions, as the features are non-specific. There is a high chance of misdiagnosis as clinical features of many common diseases can also be seen in cerebellar Infarction and therefore, it demands proper investigation [4]. Stroke not being considered in the initial diagnosis increases the risk of subsequent and more severe ischaemic events leading to complications like space-occupying oedema formation, obstructive hydrocephalus, and direct compression of the midbrain and pons [6]. Failure of proper diagnosis and early treatment may increase the overall morbidity and mortality and lead to the denial of quality care to the patients by subjecting them to unnecessary investigations and interventions.

Cerebellar infarction being in the rare category of stroke, its misdiagnosis has not been given much importance in the available literature. Accurate data on the proportion of misdiagnosed cases of cerebellar infarcts and data regarding the outcome of such patients are still not available. This review article will be analysing the various factors that contribute to the misdiagnosis of cerebellar infarction. By analysing different case reports of cerebellar infarction, the most common presentations that were under-evaluated and their subsequent outcomes will be discussed, thereby promoting the importance of proper diagnosis and reporting of cases of cerebellar infarction in the future.

Method

An extensive PubMed database search was conducted. Case reports and studies using the keywords "cerebellar ischemia", "cerebellar stroke", and "misdiagnosis of stroke" were selected from all the currently available literature in the database. Studies from different regions were included for wider representation. Study participants of all ages and articles in all the available languages published between 2000 and 2020 were included. Animal studies were excluded. Forty-nine articles were selected for the review. The articles were assessed and discussed to bring out the factors leading to misdiagnosis, various presentations, and outcomes.

## Review

Factors leading to misdiagnosis of cerebellar infarction

Misdiagnosis of cerebellar infarction can be attributable to diverse factors and determining those factors could be essential for improved diagnosis of the condition by medical professionals in the future (Table 1). Physicians are less vigilant of cerebellar infarction, as it accounts for only 2% of all ischemic strokes [2,7]. According to a study by Pandian et al., patients who presented to the emergency department late, after an episode of stroke, were unaware of the warning signs of stroke, especially the elderly. Young, educated individuals have better knowledge and self-recognition of stroke compared to the elderly population [8].

**Table 1:** Factors leading to misdiagnosis of cerebellar infarction NIHSS: National Institutes of Health Stroke Scale

Factors
Elderly people unaware of warning signs of stroke
Absent neurological signs in cerebellar stroke cases
Less reliance on clinical signs by physicians
Low NIHSS score seen in large cerebellar stroke
Absence of vascular risk factors in young
Initial negative CT findings
Lack of access to a neurologist

Masuda et al. investigated 32 misdiagnosed and 82 correctly diagnosed cerebellar infarction patients and compared the neurological symptoms like vertigo and headache with deficits like disturbance of consciousness, dysarthria, nystagmus, and ataxia. There was no difference in the prevalence of vertigo or headache between the correctly and misdiagnosed patients. But neurologic deficits, such as dysarthria, were less seen in misdiagnosed patients compared to correctly diagnosed cases [9]. Symptoms such as dysmetria, abnormal extraocular movements, nystagmus, gait abnormality and focal motor weakness neither contributed to early diagnosis nor delayed the diagnosis [10]. Calic et al. found that neurological signs are commonly absent in patients with isolated cerebellar infarction (OR 4.0, 95%CI 1.2-13.3, p = 0.03) and they also found that emergency department physicians detected fewer neurological signs compared to neurology physicians (a mean of one versus two signs, p < 0.001) [11]. Misdiagnosis of 34% of cerebellar infarction was seen, with peripheral vestibulopathy as the most common alternative diagnosis. A study conducted in Tokyo by Masuda et al. concluded that out of 32 misdiagnosed cases of cerebellar infarction, 30 were seen by physicians who were not neurologists [9]. Coordination, gait, and eye movements, which are major components of neurologic examinations to detect cerebellar strokes, are often not performed in a primary care setting, especially if the symptoms do not suggest central nervous system (CNS) cause [12]. The National Institutes of Health Stroke Scale (NIHSS), which is used to assess stroke severity focuses heavily on the deficits of anterior circulation stroke like aphasia, neglect, motor, and sensory disturbances. A very low NIHSS score can be seen in large cerebellar stroke and lead to the missing of posterior circulation strokes compared to anterior strokes [13].

In young patients without vascular risk factors, cerebellar infarction was initially misdiagnosed as migraine, gastritis, or vestibular neuritis when the clinical examination was incomplete and investigated only by cranial computed tomography (CT) [14]. Although cranial CT is the most common investigative modality employed by physicians in a hospital setting, the result is mostly unreliable and inaccurate. Images obtained in the initial 48 hours may appear normal in an ischemic stroke and bony artefacts in the posterior fossa may hide the lesion [15,16]. Masuda et al. reported that 24 out of 32 misdiagnosed cases showed a negative CT finding initially [9].

As the overall incidence of cerebellar stroke is less compared to other strokes, its diagnosis is often missed by physicians. Although the elderly population are more susceptible to stroke, their knowledge regarding the warning signs and symptoms is inadequate, leading to a late presentation to the emergency department. On the other hand, better awareness is found among young and educated individuals. Cerebellar infarction mostly presents with nonspecific symptoms like headache, nausea, vertigo, vomiting, and dizziness, making the diagnosis more difficult. But certain neurological deficits like dysarthria, ataxia, and nystagmus contribute to its early diagnosis. Also, a pure cerebellar stroke may not present with specific neurological symptoms. Hence, patients presenting to the emergency department with nonspecific symptoms have a lesser chance of receiving early treatment compared to those who present with neurological deficits specific to posterior brain strokes. Such nonspecific symptoms also lead to alternate diagnoses such as peripheral vestibulopathy, migraine, gastritis, vestibular neuritis, etc, where peripheral vestibulopathy is the most common.

Radiological diagnosis of cerebellar infarction

The advent of technology, such as radiological investigations, has made the definitive diagnosis of cerebellar infarction much easier. Magnetic resonance imaging (MRI) is the imaging modality of choice in diagnosing cerebellar infarction, but cranial CT is needed for the immediate and early diagnosis of cerebellar haemorrhage. Even though a CT scan is often the first investigation done in a clinical setting, during the early hours after the onset of an acute ischemic stroke, CT often shows a negative result especially in a posterior fossa stroke [17,18]. CT being an investigation of relatively low sensitivity, a case of cerebellar infarction presenting with atypical symptoms may be detected only with MRI [19].

In a study by Simmons et al. comparing CT and MRI, only half of the cases of cerebellar infarction were visible in CT [18]. In a similar study by Rosi et al., CT scans showed cerebellar infarction in 78% of the patients [20]. In a study conducted on 115 cerebellar infarction patients, CT was found to be abnormal only in 52 out of 115 patients (45.2%), but among 99 MRI imagings, all were positive irrespective of the time of imaging from the onset of symptoms [12]. MRI proved to be more sensitive than CT in the above three studies. Infarcts can be detected with diffusion-weighted imaging (DWI) within a few minutes from the onset but are not a reliable indicator as cytotoxic brain oedema can peak in three days [21,22]. At around three days after onset, the brain swelling enlarges, and around 10 days, infarct becomes isointense [22]. Although fluid-attenuated inversion recovery (FLAIR) and T2 weighted images (T2WI) become hyperintense a few hours from the onset, T2WI is more accurate compared to FLAIR images as the latter often gives false negatives in the posterior fossa due to local field heterogeneities [23]. Therefore, MRI guarantees accurate diagnosis when taken as soon as possible. Also, in another study, diffusion MRI was found to be useful in predicting the poor outcome of cerebellar infarction [24].

Although the importance of MRI cannot be emphasized enough, one cannot ignore its drawbacks. In a study by Kattah et al., MRI when performed within 48 hours of onset was found to show false negative results [25]. Small cerebellar infarcts are often found as incidental findings on MRI but not found during the symptomatic/acute stage because of mild symptoms and failure to perform imaging at the desired time [26]. It is difficult to detect small infarcts even in MRI in the acute stage unless there are multiple cerebellar infarctions or a concurrent infarct elsewhere in the brainstem or cerebrum [27]. Also, FLAIR and DWI images are less sensitive in posterior circulation strokes mainly due to magnetic field heterogeneities in the posterior fossa [26,28,29]. A bedside oculomotor test, the head impulse-nystagmus-test of skew (HINTS), promises a more accurate diagnosis of cerebellar stroke in patients with an initial negative MRI. In a study by Kattah et al. in acute vestibular syndrome patients, where initial MRI had shown a false negative result in 12% of cases, HINTS showed an overall sensitivity of 100% and specificity of 96% for diagnosing stroke. This also included patients with pure cerebellar stroke [25]. Also, in six other independent studies, a positive HINTS test increased the risk of posterior circulation stroke by 15 times. Here, the sensitivity was 95.5% and the specificity was 71.2%. HINTS was found to be efficient in differentiating posterior circulation stroke from the peripheral causes of acute vestibular syndrome. A thorough clinical examination along with imaging techniques is the best practice to detect and efficiently manage cerebellar infarctions.

In this era of constantly advancing technologies and incorporation of artificial intelligence in imaging modalities, it is more likely that diagnosis of various diseases centres around imaging investigations rather than comprehensive clinical examination. Diagnosis of cerebellar infarctions is also no different and this over-dependence on radiological investigations has often led to misdiagnosis. CT, being the immediate and most affordable investigation, is more suitable for haemorrhages but yields negative results, especially in posterior fossa infarctions. MRI is the imaging modality of choice and studies have shown that MRI is more sensitive in detecting cerebellar infarctions compared to CT. DWI and FLAIR can detect cerebellar infarction within minutes, with the former being more accurate and the timing from the onset of symptoms is important since the brain oedema can peak within three hours. Therefore, care should be taken that investigations are conducted and repeated if required, keeping in mind the possibility of worsening later. MRI can also be helpful in predicting the poor outcome of cerebellar strokes, apart from its diagnostic value. Even though MRI has high sensitivity, the non-specific symptoms of cerebellar infarction prevent physicians from ordering an MRI. Also, in many cases of small infarcts, there is a higher chance that the findings are missed even if an MRI is done, unless there are multiple infarcts. Some other common drawbacks of MRI are the availability (lack of MRI capacity in smaller hospitals), high cost of MRI, especially in resource-limited and self-pay settings, inability to get MRI done in a specific subset of patients (such as metal in the body or unstable patients from cardiorespiratory standpoint who may not be able to lay flat for a prolonged period for MRI). The significance of clinical examination can be reinforced in such cases, as studies have proved that HINTS, a bedside test has more sensitivity and specificity than imaging for posterior circulation strokes, and can be used to rule out peripheral causes of acute vestibular syndrome. But further management of the patient necessitates further investigations in addition to the clinical findings. Therefore, it is imperative that clinical examination and advanced technologies are incorporated together into the diagnosis of cerebellar infarctions. The presenting symptoms, time of presentation, infrastructure and facilities of the health centre, the efficiency of the medical professionals, and many other contributing factors together decide the diagnostic procedure. Clinical skills and imaging techniques should be balanced and utilised in supporting one another.

Association of misdiagnosis with clinical characteristics

Understanding the relationship between various clinical presentations of cerebellar infarction and its misdiagnosis is crucial. While some presentations lead to an early and accurate diagnosis, some symptoms being non-specific delays the diagnosis even further. Even though the presenting symptoms in cerebellar infarction are often dizziness, gait disturbance, nausea and vomiting, a study among 115 cerebellar infarction patients showed that misdiagnosis is often related to nausea and vomiting (OR 2.3, 95%CI 1.01-5.5, p = 0.046), absence of neurological signs (OR 3.5, 95%CI 1.5-8.0, p = 0.003), and isolated cerebellar infarction (OR 2.2, 95%CI 1.01-4.8, p = 0.047). Whereas dysarthria (OR 3.9, 95%CI 1.6-9.6, p = 0.003) and hemiparesis (OR 0.3, 95%CI 0.1-0.9, p = 0.04) led to confirmation of the diagnosis of cerebellar infarction [11]. In a study done in the United States on 465 patients with missed ischemic stroke, symptoms relating to posterior circulation strokes had a greater chance of misdiagnosis [13]. A stroke was more likely to be missed with symptoms like nausea/ vomiting (OR 4.02, 95% CI 1.60-10.1) and dizziness (OR 1.99, 95%CI 1.03 - 3.84). Also, symptoms like focal weakness (OR 0.396, 95%CI 0.228-0.688), dysarthria (OR 0.048, 95%CI 0.288-0.004), vision changes (OR 0.377, 95%CI 0.176-0.809) had a lower chance of missed diagnoses.

In a study which included 32 (28%) misdiagnosed cerebellar infarctions, it was found that dysarthria usually seen in the medial branch of posterior inferior cerebellar artery territory infarction is associated with a lesser chance of misdiagnosis. Neurologic symptoms like vertigo and headache were equally prevalent in both misdiagnosed and correctly diagnosed patients [9]. In a similar study by Sangha et al. in 47 confirmed cases of cerebellar infarction, it was found that misdiagnosis was least associated with weakness (10.7%) and change in speech (21.4%), while most frequently seen in vertigo (64.4%) [10]. Uraguchi et al., in a study done with 250 cases of posterior circulation stroke, reported that eight cases that presented with symptoms like dizziness and dysphagia and a negative MRI-DWI were initially missed and later diagnosed when referred to the department of otolaryngology [30]. In a study by Lee SH et al., 11% (four out of 35 cases) of patients with primary cerebellar haemorrhage were misdiagnosed [31]. These patients had presented either with gastrointestinal symptoms or hypertension. Also, misdiagnosis was associated with a normal mental state (100% vs 35%, p= 0.07) and nausea or vomiting (100% vs 58%, p= 0.22). Venkat et al. stated that, among the 141 ischemic and 15 hemorrhagic strokes that were misdiagnosed, posterior circulation strokes were more common and that altered mental status, nausea or vomiting, dizziness and vertigo, symptom resolution were associated with a greater chance of misdiagnosis (p<0.05). On the other hand, symptoms such as dysarthria and hemiparesis aided in an accurate diagnosis (p<0.05) [32].

Posterior circulation strokes, mainly those affecting the anterior inferior cerebellar artery present with symptoms similar to that of inner ear disease. Therefore, misdiagnosis is common when presented with such overlapping symptoms [33]. Compared to anterior circulation, misdiagnosis is three times more common in posterior circulation strokes as it is associated with atypical symptoms. Nausea/vomiting (OR 4.02; 95%CI 1.60-10.1), dizziness (OR 1.99; 95%CI 1.03-3.84), and a positive stroke history (OR 2.40; 95%CI 1.30-4.42) were more likely to be misdiagnosed [13]. In a study by Chen et al., posterior circulation infarcts presenting as isolated vertigo were more frequently misdiagnosed as peripheral vertigo [34]. Misdiagnosis is more frequent with mild, non-specific and transient symptoms. This suggests that symptom-specific factors contribute to misdiagnosis. Table 2 lists the symptoms of cerebellar infarction that can lead to misdiagnosis.

**Table 2:** Misdiagnosed symptoms in cerebellar infarction

Symptoms often misdiagnosed	Symptoms less likely misdiagnosed
Vertigo	Disturbance of consciousness
Headache - migraine	Dysarthria
Gastrointestinal symptoms - gastritis	Focal weakness
Hypertension	Nystagmus
Nausea, vomiting	Ataxia
Dizziness, gait disturbance	Visual changes

The non-specificity of symptoms and presentation of cerebellar infarction has always been a factor leading to misdiagnosis. At times it is possible to detect cerebellar strokes instantly from the presentation whereas at other times it can be totally confusing. In many of the similar studies on misdiagnosed cerebellar infarction, nausea and vomiting were found to be associated with increased chances of misdiagnosis. Likewise, patients presenting with symptoms of posterior circulation stroke were more frequently misdiagnosed compared to anterior circulation. The absence of neurological signs and isolated cerebellar infarction were other associations of delayed diagnosis. However, the diagnosis was easier in cases with dysarthria and hemiparesis. The presence of vision changes, signs of focal deficit, and dysarthria reduced the chances of misdiagnosis. Many of the studies were homogenous of these findings but there were also certain studies that stated that neither early diagnosis nor misdiagnosis/late diagnosis is associated with dysmetria, abnormal extraocular movements, nystagmus, gait abnormality and focal motor weakness. Vertigo and headache were some of the symptoms which are equally seen both in misdiagnosed and correctly diagnosed cases of cerebellar infarction. Rare presentations with gastrointestinal symptoms have also been reported that have greater chances of being misdiagnosed. Isolated vertigo and other symptoms of inner ear disease have often masked the diagnosis of cerebellar stroke and are mistaken to be peripheral vertigo. Heterogenous conclusions were derived from the studies regarding the association between clinical characteristics and misdiagnosis, but the majority of the studies were in accordance with the conclusion that nausea and vomiting are associated with increased misdiagnosis, whereas hemiparesis and dysarthria helped to arrive at an accurate diagnosis. Clinical features have a role but are not solely responsible for the misdiagnosis. Meticulous evaluation of the patient with investigatory support can, to a large extent, improve the scenario of misdiagnosis.

Prognosis, outcome, and rehabilitation of cerebellar infarction

Learning the outcome, complications, and prognosis of a misdiagnosed cerebellar stroke emphasises the importance of its accurate and early diagnosis. A five-year study of 15 misdiagnosed cases of cerebellar infarction revealed an overall mortality of 40%, and among the survivors, 50% had persistent disabling deficits. The reason for death included brainstem compression and disabling defects including gait abnormalities [35]. In a study done on 106 cerebellar stroke patients, two out of 15 patients who developed hydrocephalus died. Also, the mean hospital stay was longer for patients with complications [36]. Cerebellar strokes are often misdiagnosed as vertigo and result in failure of diagnostic stroke workup and absence of secondary prevention. This can increase the risk of recurrence of cerebellar infarctions. In addition to recurring stroke, it can also progress to brain stem Infarction [31]. Also, vertigo being a non-specific symptom has a poorer prognosis when it is associated with cerebellar infarction than vertigo due to other causes [9]. A study by Ng et al. on 79 cerebellar stroke patients concluded that cerebellar haemorrhages have a poorer prognosis compared to cerebellar infarction, as cerebellar haemorrhages can act as a space-occupying lesion that results in a coma or apneic state. They often need rapid decompression by craniectomy [37]. However certain studies showed that the risk of complications is not significantly (p=0.57) correlated with misdiagnosis. Length of hospital stay may be extended but only two out of 114 patients developed complications such as cerebral oedema and hydrocephalus in a study by Calic et al. [11]. Initial correct diagnosis and timely management possess a greater advantage over misdiagnosis, irrespective of the disease. In a study by Kelly et al., it was noted that survivors of new cerebellar stroke in admission to rehabilitation centres showed excellent functional recovery; 74% of the patients were treated medically and 26% were treated surgically during rehabilitation [38].

Ioannides et al. noted that cerebral oedema and death of the patient can follow the misdiagnosis and delayed treatment of cerebellar infarction, whereas it can be limited to functional debility and acute complications of hospitalization such as venous thromboembolism, pressure ulcer, urinary tract infections, and pneumonia if managed earlier [4]. Cerebellar strokes when managed with optimal care and treatment such as decompressive surgery promises favourable outcome. Mass effects due to large infarctions if treated promptly and closely monitored reduces complications such as brain stem displacement, obstructive hydrocephalus and even death [5]. Balance, muscle strengthening and gait-focused physical therapy has been found to be effective in reducing the severity of cerebellar ataxia. Being time-restrictive, further studies are required to assess the long-term outcomes [39]. The advancements of the modern era should be channelled to aid the accurate diagnosis and further management of cerebellar stroke.

Cerebellar stroke although rare, results in unfavourable outcomes if not managed early. While the overall mortality is 40%, 50% of the survivors have permanent disabling deficits (Table 3). The complications of cerebellar stroke range from gait abnormality to serious outcomes like hydrocephalus and brain stem compression. Very often, cerebellar stroke presents as vertigo. This contributes to a delayed diagnosis that may later progress to a brain stem Infarction. A recurrence of the stroke can also be expected. It is found that vertigo as a symptom when caused by cerebellar stroke has a poor prognosis compared to when it occurs due to other diseases. Haemorrhage in the cerebellum, when not treated promptly, can lead to coma and an apneic state. While the majority of the studies on cerebellar infarction and its outcome concluded that an early and accurate diagnosis contributes to a better prognosis and less severe complications, few studies have found that there is no significant relationship between early diagnosis and outcome. However, interventions like rapid decompression by craniectomy may contribute to a better outcome. Although time restrictive, physiotherapy may be considered in improving the long-term complications of cerebellar stroke like gait abnormality, reduced muscle strength, and impaired balance.

**Table 3:** Impacts of cerebellar stroke misdiagnosis

Outcomes
Development of complications like brain oedema, obstructive hydrocephalus
Poor prognosis/outcome among patients
Decreased quality of life
Increased morbidity and mortality
No proper stroke workup and secondary prevention - high risk of recurrent stroke

## Conclusions

Cerebellar strokes associated with high mortality or morbidity often present with overlapping and non-specific symptoms and if misdiagnosed can progress to severe complications such as brain oedema and obstructive hydrocephalus. A large number of cerebellar infarctions have been misdiagnosed owing to various factors and this can have an adverse effect on the outcome of the patient. Lesser incidence, unawareness of the warning signs, overlapping non-specific symptoms, lack of neurological symptoms, failure to perform a detailed neurological clinical examination, and overdependence on imaging investigations have all been accountable for the misdiagnosis of cerebellar infarctions. A combination of the necessary neurological examinations and appropriate radiological investigations provide a better and early diagnosis of cerebellar stroke.

The importance of clinical examination cannot be emphasized enough as even the latest radiological imaging techniques are not free of flaws. Also, a thorough knowledge of the association between various clinical presentations of cerebellar stroke and its misdiagnosis helps clinicians to be more vigilant about the disease. Cerebellar stroke may be a rare disease but the complications following an episode may be life-threatening. Understanding the importance of an early accurate diagnosis and management, with an attempt to improve the quality of the patient's life is the need of the hour in addition to a better prognosis and lesser complications.
